# Association between self-reported discrimination and body weight dissatisfaction among adolescents in a city in Santa Catarina, Brazil

**DOI:** 10.1590/1984-0462/2026/44/2025190

**Published:** 2026-06-22

**Authors:** Melquesedek Ferreira da Silva Almeida, Suellem Zanlorenci, Diego Augusto Santos Silva

**Affiliations:** a Universidade Federal de Santa Catarina, Florianópolis, SC, Brazil.

**Keywords:** Bullying, Sexism, Dysphoria, Health risk behaviors, Prejudice, Bullying, Sexismo, Disforia, Comportamento de risco a saúde, Prejuízo

## Abstract

**Objective:**

This study aimed to examine the association between body weight dissatisfaction and self-reported experiences of discrimination among Brazilian adolescents, stratified by sex.

**Methods:**

Data were obtained from the "Brazilian Guide for the Assessment of Physical Fitness and Lifestyle Habits-Stage II", conducted in 2019 with a city-representative sample of school adolescents aged 14–17 years from São José, Santa Catarina, Brazil. Body weight dissatisfaction and experiences of discrimination were measured using standardized instruments and through self-report. Weighted prevalence estimates and sex-stratified logistic regression models were used to evaluate associations, adjusting for sociodemographic variables.

**Results:**

In the adjusted analysis, adolescents of both sexes who reported discrimination had higher odds of wanting to increase their body weight (odds ratio — OR 2.05; 95% confidence interval — 95%CI 1.41–2.98) and to decrease their body weight (OR 1.42; 95%CI 1.09–1.85). Stratified analyses revealed different patterns by sex: among male adolescents, discrimination was associated with higher odds of wanting to decrease body weight (OR 1.66; 95%CI 1.58–1.74), whereas among female adolescents, discrimination was associated with higher odds of wanting to increase body weight (OR 3.02; 95%CI 1.72–5.31).

**Conclusions:**

Dissatisfaction with body weight and experiences of discrimination are prevalent among Brazilian adolescents, with distinct patterns between boys and girls. Discrimination was associated with both the desire to gain and to lose weight, but in sex-specific directions, suggesting that social pressures shape body image differently for male and female adolescents.

## RESUMO

**Objetivo:**

Verificar a prevalência de satisfação/insatisfação com o peso corporal e associação com discriminação autorrelatada entre adolescentes, estratificados por sexo.

**Métodos:**

Os dados foram obtidos do “Guia Brasileiro de Avaliação da Aptidão Física e Hábitos de Vida – Etapa II”, conduzido em 2019, com amostra representativa de adolescentes escolares de 14 a 17 anos do município de São José, Santa Catarina, Brasil. A insatisfação com o peso corporal e as experiências de discriminação foram mensuradas por instrumentos padronizados e autorrelato. Utilizaram-se modelos de regressão logística multinomial, com análises estratificadas por sexo, ajustadas para variáveis sociodemográficas.

**Resultados:**

Na análise ajustada, adolescentes de ambos os sexos que relataram discriminação apresentaram maior probabilidade de desejar aumentar o peso corporal (odds ratio — OR 2,05; intervalo de confiança de 95% — IC95% 1,41–2,98) e de desejar diminuir o peso corporal (OR 1,42; IC95% 1,09–1,85). As análises estratificadas revelaram padrões distintos segundo o sexo: entre os meninos, a discriminação esteve associada a maior probabilidade de desejar reduzir o peso corporal (OR 1,66; IC95% 1,58–1,74), enquanto entre as meninas esteve associada a maior probabilidade de desejar aumentar o peso corporal (OR 3,02; IC95% 1,72–5,31).

**Conclusões:**

A insatisfação com o peso corporal e a discriminação autorrelatada são prevalentes entre adolescentes, apresentando padrões distintos entre os sexos. A discriminação associou-se tanto ao desejo de ganhar quanto ao de perder peso, em direções específicas segundo o sexo, sugerindo que pressões sociais moldam a satisfação e a insatisfação com o peso corporal de forma diferente entre adolescentes do sexo masculino e feminino.

## INTRODUCTION

 Body weight perception and nutritional status represent fundamentally different dimensions and should be analyzed separately.^1^ While nutritional status is an objective indicator derived from standardized anthropometric measures, weight perception is a subjective interpretation shaped by psychological, social, and cultural factors. Consequently, adolescents may perceive themselves as underweight or overweight regardless of their actual condition, which reinforces the importance of investigating body weight perception and the desire to change it.^1^ Research conducted in European and North American nations estimated that body weight dissatisfaction among teenagers ranges from 37.7% in the United States of America (USA) to 39.9% in Italy, for men, and from 51.0 to 55.2% in Italy and the USA, respectively, for women aged 11–15 years.^2^ In Brazil, a 2014 prevalence study among adolescents aged 12 to 17 years estimated that 45.0% were dissatisfied with their body weight.^3^ In general, factors such as negative weight-related comments and teasing are common among adolescents and can influence body weight satisfaction.^4,5^

 Discrimination negatively impacts both mental and physical well-being of those exposed to it, causing stress and social trauma,^5^ which may influence body weight satisfaction. Adolescents exposed to discrimination are more likely to engage in suicidal behavior or binge eating, and exhibit reduced mental and physical well-being, shame, and chronic pain.^6^ Furthermore, the adverse and long-lasting effects of discrimination stem from multiple interpersonal sources, including peers, family, and educators.^7^ These experiences can manifest in various forms, like weight discrimination, racial/ethnic discrimination, xenophobia, and discrimination based on sex or gender.^8^

 Body appearance is a common target of discrimination, a phenomenon referred to as body shaming, which is particularly prevalent among adolescents and typically targets individuals whose bodies deviate from societal standards.^9^ Discrimination can act as a stressor that contributes to weight gain, especially among females, who may experience societal pressures that contrast with Western beauty ideals.^10^ Additionally, discriminatory actions like teasing and bullying are increasingly common among adolescents of both genders.^7^

 A cohort study of Australian adolescents aged 14 to 15 years, whose families were recruited when the child was four or five years of age and interviewed every two years, revealed that adolescents who experienced body-related discrimination had higher probability of reporting fear of gaining weight, loss of control over eating, skipping meals, using medication or vomiting to control weight, engaging in restrictive eating, and exercising to manage weight.^11^ Future studies should aim to identify protective strategies to mitigate the adverse health effects of discrimination.^4^

 To gain a deeper comprehension of the association between discrimination and body dissatisfaction, a theoretical model was proposed, mapping discrimination experiences to the internalization of weight bias, body weight dissatisfaction, and five symptoms, including binge eating, purging, restriction, excessive exercise, and muscle-building behaviors.^12^ The consequences of discrimination include behavioral disorders associated with peer problems, chronic emotional distress, low self-esteem, risky sexual behavior, poor educational outcomes, low professional engagement, and the application of harmful substances and products.^13^

 Although the literature recognizes that self-reported discrimination impacts body weight dissatisfaction and promotes habits that affect body composition, discrimination is also associated with psychological aspects of physical activity, including greater motivation to avoid exercise, lower self-efficacy, and reduced motivation to engage in physical activity when internalized.^14^ Similar associations between weight discrimination and health outcomes have also been observed.^11^

 However, evidence from South American countries is scarce; therefore, investigating factors related to body weight dissatisfaction among South American adolescents can fill an important gap in the literature and expand our understanding of a phenomenon that has been largely documented in high-income countries, particularly considering social and contextual determinants that may shape this relationship in understudied populations.^15^ This study aimed to examine the association between self-reported experiences of discrimination and body weight dissatisfaction among adolescents from public schools in a city in southern Brazil, stratified by sex. 

## METHOD

 This was a cross-sectional investigation conducted with adolescent students aged 14 to 19 years from a city in southern Brazil. This study was conducted and reported following the Strengthening the Reporting of Observational Studies in Epidemiology (STROBE) guidelines. The study is part of the larger project titled "Brazilian Guide for the Assessment of Physical Fitness and Lifestyle Habits — Stage II", conducted in São José, Santa Catarina, Brazil. In 2019, the population of high school students enrolled in the city’s state public schools was 5,411. 

 To estimate the sample size, a two-stage cluster sampling plan was developed. In the first stage, school density (small, fewer than 200 students; medium, 200 to 499 students; and large, 500 or more students) was used as the stratification criterion. The city had 11 eligible schools, which were selected proportionally by size. Within the second stage, study shift and grade level were considered. The 11 eligible schools comprised 186 high school classes. All students in the selected classes were invited to participate in the research. 

**Figure 1:**
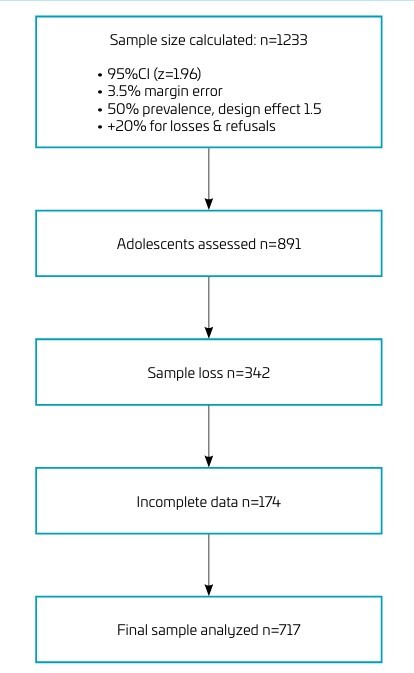
Strengthening the Reporting of Observational Studies in Epidemiology (STROBE) flowchart of the study sample selection process

 To determine the sample size, the following parameters were adopted: a confidence level of 1.96 (95% confidence interval), a tolerable absolute error of 3.5 percentage points, a prevalence of 50% (unknown outcome), and a design effect of 1.5. An additional 20% was included to account for potential losses and refusals. Based on these parameters, the required sample size was 1,233 students. However, the project assessed 891 adolescents from public schools, resulting in a 27% sample loss. Of the total number of adolescents evaluated, 717 had complete information on the variables of the current research, constituting the study sample (Figure 1). Additional methodological details of the larger project are published elsewhere.^16^

 The Federal University of Santa Catarina Human Research Ethics Committee approved this study under protocol number 3.523.470, Certificate of Presentation for Ethical Appreciation — CAAE: 17042019.2.0000.0121. All parents or guardians signed the Informed Consent Form authorizing adolescents under 18 years of age to participate in the research. In addition, all adolescents signed the Assent Form, providing consent to participate in the research. Body weight perception is subjective and strongly influenced by social and cultural norms. In this study, body weight satisfaction was conceptualized as the adolescents’ perception that their current weight status was appropriate or desirable.^14^ Conversely, body weight dissatisfaction refers to a perceived discrepancy between actual and desired body weight, which may manifest as a desire to increase or decrease it. Body weight dissatisfaction was assessed using the Youth Risk Behavior Surveillance System, which was validated in the Brazilian population.^17^ Adolescents responded to the subsequent question, "How would you describe your body weight?", with the following possibilities for responses: "much thinner than I expect", "a little thinner than I expect", "about as I would like", "a little larger than I expect", or "much larger than I expect". The responses "much thinner than I expect" and "a little thinner than I expect" were categorized as "dissatisfied due to thinness". The response "about as I would like" was defined as "satisfied with body weight", and "a little larger than I expect" and "much larger than I expect" were combined and categorized as "dissatisfied due to overweight".^15^ This was the dependent variable. The reproducibility of these questionnaire items is high (kappa agreement index=0.84), supporting their application in Brazilian epidemiology studies.^17^

 Self-reported discrimination was assessed using the subsequent query: 

 "In the past 12 months, have you felt discriminated against for the following reasons:

- skin color or race;
- religion or worship;
- being poor or rich;
- illness or physical disability;
- being overweight;
- being underweight;
- the way you dress;
- other characteristics".

Response options for each question were "yes" or "no". For the "other characteristics" option, participants could specify the reason in writing. Self-reported discrimination was dichotomized as "no" or "yes", where a positive response to any of the listed reasons was classified as "yes" (i.e., self-reported discrimination, the independent variable). Conversely, if participants responded "no" to all options, they were categorized as "no" for this variable. This approach has been used in other surveys on self-reported discrimination among adolescents.^5^

 The following covariates were included in this study: age, skin color/race, purchasing power [assessed using a questionnaire widely applied in Brazil to classify family assets, generating scores that range from category E (lowest) to A (highest); in this study, categories A, B1, and B2 were grouped as higher purchasing power, whereas categories C1, C2, D, and E were classified as lower purchasing power], and sexual maturation (assessed according to Tanner’s criteria and categorized as prepubertal, pubertal and postpubertal according to the recommendations in the literature).^18^ Information on lifestyle behaviors was collected using the Fantastic Lifestyle Questionnaire, which assesses behaviors during a typical week and was validated in the Brazilian population.^19^ Dietary habits were evaluated using the question "Do you eat a balanced diet?", referring to eating habits over the previous seven days; responses "almost never", "rarely", and "sometimes" were categorized as inadequate, whereas "relatively often" and "almost always" were categorized as adequate. Alcohol use was assessed by the question "During the last 30 days, on how many days did you have five or more alcoholic drinks on the same occasion?", with responses categorized as "yes" (one day, two days, three to five days, six to nine days, 10 to 19 days, or 20 or more days) and "no" (no days). Smoking was assessed using the question "Do you smoke cigarettes?", with responses categorized as "yes" (more than ten cigarettes per day or one to ten per day) and "no" (none in the last six months, none in the last year, or never smoked). Body mass index (BMI) was later categorized according to sex and age into "thinness,", "normal weight,", "overweight," and "obese," in accordance with recommendations from the World Health Organization, while physical activity was verified through a questionnaire question Youth Risk Behavior Survey (YRBS),^17^ through the following question: During a normal (typical) week, on how many days do you practice moderate to vigorous physical activity? The answers were categorized as follows: physically active adolescents who accumulate at least 60 minutes of daily moderate to vigorous physical activity on five or more days of the week. These variables were considered because of their associations with body weight dissatisfaction and perceived discrimination. 

 Inferential and descriptive statistics were used. Categorical variables were presented as relative frequencies (%), with 95% confidence intervals (95%CI). Additionally, the heterogeneity chi-square test was used to identify potential variations among groups based on body weight satisfaction or dissatisfaction. Considering the determining role of sex in shaping an individual’s perception of body weight, and the fact that factors associated with body weight satisfaction/dissatisfaction differ by sex,^20^ all analyses were stratified accordingly. 

 Multinomial logistic regression was used to examine associations among the variables in this study. The regression coefficients were exponentiated to estimate relative risk ratios (RRRs), which, in cross-sectional studies, are interpreted similarly to odds ratios (ORs). Accordingly, we present ORs and 95%CIs, using "satisfied with body weight" as the reference category, to test the association between self-reported discrimination and body weight satisfaction/dissatisfaction. Each multinomial regression model included age, purchasing power, unhealthy lifestyle habits (poor diet, physical inactivity, alcohol consumption, and smoking), sexual maturation, and BMI as control variables. 

 The analyses accounted for sample weights and the survey design. All statistical analyses were conducted using Stata software (version 16.0, StataCorp LP, College Station, TX, USA), with a significance level set at p<0.05. 

## RESULTS

 This study evaluated 717 adolescents (345 or 48% were males). Among women, 80.38% expressed dissatisfaction with their body weight, with 25.27% dissatisfied due to thinness and 55.11% due to being overweight. Among men, 67.82% were dissatisfied with their body weight, with 32.17% dissatisfied due to thinness and 35.65% due to being overweight (Table 1). 

**Table 1:** General characteristics of the sample and according to sex. São José, SC, Brazil – 2019.

Variables		Total sample		Male		Female		p-value
		n	%	n	%	n	%	
		717	100	345	48.12	372	51.88	
Body weight								
	Satisfied with body weight	184	25.66	111	32.17	73	19.62	**0.033**
	Dissatisfied with thinness	205	28.59	111	32.17	94	25.27	
	Dissatisfied with excess weight	328	45.75	123	35.65	205	55.11	
Self-reported discrimination								
	Reported feeling discriminated against	329	45.89	129	37.39	200	53.76	0.109
	Reported not feeling discriminated against	388	54.11	216	62.61	172	46.24	
Age (years)								
	14–16	321	44.77	150	43.48	171	45.97	0.574
	17–19	396	55.23	195	56.52	201	54.03	
Purchasing power								
	High power	464	64.71	232	67.25	232	62.37	0.045
	Low power	253	35.29	113	32.75	140	37.63	
Smoking								
	Not exposed	586	81.73	285	82.61	301	80.91	0.844
	Exposed	131	18.27	60	17.39	71	19.09	
Alcohol abuse								
	Not exposed	419	58.44	207	60.00	212	56.99	0.834
	Exposed	298	41.56	138	40.00	160	43.01	
Balanced diet								
	Suitable	112	15.62	61	17.68	51	13.71	0.338
	Inadequate	605	84.38	284	82.32	321	86.29	
Physically active								
	Yes	63	8.79	40	11.59	23	6.18	**0.033**
	No	654	91.21	305	88.41	349	93.82	
Sexual maturation								
	Prepubescent	74	10.32	34	9.86	40	10.75	0.805
	Pubescent	488	68.06	234	67.83	254	68.28	
	Post pubertal	155	21.62	77	22.32	78	20.97	
BMI status								
	Normal	502	70.01	249	72.17	253	68.01	**0.003**
	Overweight/Obesity	215	29.99	96	27.83	119	31.99	

n: frequency number, %: percentage; BMI: body mass index; EL: economic level; LPA: level of physical activity.

Notes: p-value in bold means<0.05.

 Among adolescents of both genders who reported feeling discrimination, for females who reported feeling discriminated against, 86% were dissatisfied with the weight of their bodies, with 31% dissatisfied because of thinness and 55% because of being overweight. Among males who reported feeling discrimination, 65.89% were dissatisfied with the weight of their bodies, with 26.36% dissatisfied because of thinness and 39.53% because of being overweight. 

 According to Table 2, in the crude analysis, adolescents of both sexes who reported feeling discriminated against had a higher chance of being dissatisfied due to thinness (OR 2.09; 95%CI 1.73–2.51) and dissatisfied due to being overweight (OR 1.93; 95%CI 1.54–2.42). In the adjusted analysis, adolescents of both sexes who reported feeling discriminated against had a higher odds of dissatisfaction due to thinness (OR 2.05; 95%CI 1.41–2.98) and dissatisfaction due to overweight (OR 1.42; 95%CI 1.09–1.85), even after controlling for covariates (sex, age, skin color, purchasing power, diet, physical activity, alcohol consumption, smoking, BMI, and sexual maturation). In addition, in the adjusted analysis, female adolescents had higher odds of dissatisfaction with excess weight than male adolescents (OR 3.16; 95%CI 1.60–6.22). Regarding smoking, exposed individuals showed greater odds of dissatisfaction due to being overweight in the adjusted analysis (OR 1.76; 95%CI 1.34–2.30). Finally, overweight/obesity, assessed by BMI, was inversely associated with dissatisfaction due to thinness in the adjusted analysis (OR 0.29; 95%CI 0.24–0.34), indicating lower odds of dissatisfaction due to thinness among individuals with excess weight or obesity (Table 2). 

**Table 2:** Crude and adjusted multinomial logistic regression analysis between self-reported discrimination and body weight dissatisfaction for total sample. São José, SC, Brazil – 2019.

		Crude analysis				Adjusted analysis			
		Dissatisfied with thinness		Dissatisfied with being overweight		Dissatisfied with thinness		Dissatisfied with being overweight	
		OR	95%CI	OR	95%CI	OR	95%CI	OR	95%CI
Total sample									
Feeling of discrimination									
	Yes	2.09	(1.73–2.51)*	1.93	(1.54–2.42)*	2.05	(1.41–2.98)*	1.42	(1.09–1.85)*
Sex									
	Female	1.28	(0.86–1.92)	2.53	(1.74–3.67)*	1.17	(0.56–2.42)	3.16	(1.60–6.22)*
Age (years)									
	17–19	1.13	(3.22–3.96)	0.88	(2.30–2.68)	(2.30–2.68)	0.682	1.24	(0.81–1.91)
Skin color									
	Brown	1.18	(0.91–1.51)	0.97	(0.59–1.61)	1.12	(0.92–1.36)	1.14	(0.95–1.37)
	Black	1.81	(0.97–3.37)	0.81	(0.39–1.68)	1.68	(0.69–4.07)	0.74	(0.40–1.38)
	Yellow	1.59	(0.87–2.91)	1.36	(0.17–10.56)	1.45	(1.34–1.56)*	2.29	(0.17–30.88)
	Indigenous	0.59	(0.01–49.88)	0.26	(0.02–2.96)	0.45	(0.01–87.4)	0.23	(0.06–0.86)*
Purchasing power									
	High	0.33	(0.23–4.74)	0.71	(0.76–6.71)	0.88	(0.60–1.30)	1.43	(0.32–6.24)
Smoking									
	Exposed	0.72	(0.16–3.23)	1.29	(0.15–10,90)	1.34	(0.60–2.98)	1.76	(1.34–2.30)*
Alcohol abuse									
	Exposed	1.92	(0.34–10.65)	2.22	(0.16–2.93)	0.98	(0.44–2.20)	1.31	(0.84–2.03)
Balanced diet									
	Inadequate	0.51	(0.06–3.89)	0.39	(0.06–2.34)	0.97	(0.79–1.19)	0.80	(0.54–1.18)
Physically active									
	No	1.08	(0.15–7.76)	2.63	(0.03–10.77)	0.66	(0.12–3.18)	0.81	(0.49–1.30)
Sexual maturation									
	Pubescent	0.73	(0.05–9.10)	0.21	(0.08–5.64)	0.88	(0.16–4.75)	0.81	(0.78–0.83)
	Post pubertal	0.50	(0.06–3.73)	0.14	(0.07–3.02)	0.84	(0.08–8.60)	0.67	(0.12–3.55)
BMI									
	Overweight/Obesity	4.70	(0.60–36.20)	4.44	(0.08–24.37)	0.29	(0.24–0.34)*	11.5	(0.83–158.25)

Notes: Models adjusted for sex (male), age (14–16 years), skin color (White), purchasing power (lower economic power), diet (suitable), physical activity (not sufficiently physically active), alcohol consumption (not exposed), smoking (not exposed), body mass index (normal weight), and sexual maturation (prepubescent). The categories presented in parentheses represent the reference groups.

CI: confidence interval; OR: odds ratio; BMI: body mass index.

* p<0.05.

 According to Table 3, in the crude analysis, male adolescents who reported feeling discriminated against were more likely to be dissatisfied due to being overweight (OR 1.66; 95%CI 1.58–1.74). In the crude analysis, male adolescents of Indigenous descent were less likely to be dissatisfied with being overweight (OR 0.01; 95%CI 0.01–0.02) than the reference category (male adolescents of White skin color). In the adjusted analysis, male adolescents who reported feeling discriminated against were more likely to be dissatisfied due to being overweight (OR 1.32; 95%CI 1.08–1.62), even after controlling for covariates (age, skin color, purchasing power, diet, physical activity, alcohol consumption, smoking, BMI, and sexual maturation). In addition, in the adjusted analysis, male adolescents with Indigenous skin color had lower odds of dissatisfaction due to being overweight (OR 0.01; 95%CI 0.01–0.05), and male adolescents with overweight/obesity, assessed by BMI, had higher odds of dissatisfied due to being overweight (OR 8.39; 95%CI 0.03–2.38) (Table 3). 

**Table 3:** Crude and adjusted multinomial logistic regression analysis between self-reported discrimination and body weight dissatisfaction for males. São José, SC, Brazil – 2019.

		Crude analysis				Adjusted analysis			
		Dissatisfied with thinness		Dissatisfied with being overweight		Dissatisfied with thinness		Dissatisfied with being overweight	
		OR	95%CI	OR	95%CI	OR	95%CI	OR	95%CI
Male									
Feeling of discrimination									
	Yes	1.54	(0.68–3.47)	1.66	(1.58–1.74)*	1.65	(0.49–5.55)	1.32	(1.08–1.62)*
Age (years)									
	17–19	0.88	(0.37–2.07)	0.18	(0.02–1.33)	1.27	(0.47–5.20)	1.04	(0.37–2.90)
Skin color									
	Brown	1.09	(0.83–1.44)	0.99	(0.88–1.11)	1.04	(0.97–1.12)	1.19	(0.22–6.38)
	Black	1.43	(0.36–5.62)	0.81	(0.14–4.59)	1.41	(0.35–5.70)	0.70	(0.15–3.18)
	Yellow	1.73	(0.73–4.08)	0.26	(0.06–1.03)	2.02	(0.58–6.94)	0.21	(0.05–0.77)*
	Indigenous	0.52	(0.03–7.24)	0.01	(0.01–0.02)*	0.40	(0.01–15.38)	0.01	(0.01–0.05)*
Purchasing power									
	High	0.54	(0.04–6.25)	0.99	(0.38–2.56)	0.75	(0.19–2.94)	1.47	(0.01–21.4)
Smoking									
	Exposed	1.28	(0.18–8.90)	0.83	(0.28–3.51)	1.09	(0.32–3.64)	3.16	(0.63–15.79)
Alcohol abuse									
	Exposed	1.47	(0.29–7.34)	0.55	(0.10–3.03)	0.65	(0.09–4.71)	1.46	(0.49–4.32)
Balanced diet									
	Inadequate	0.70	(0.05–8.32)	0.34	(0.23–0.49)	1.07	(0.51–2.20)	0.88	(0.66–1.17)
Physically active									
	No	0.43	(0.02–6.35)	1.36	(0.02–7.27)	0.76	(0.11–4.99)	0.53	(0.01–29.67)
Sexual maturation									
	Pubescent	3.28	(1.76–6.13)	0.22	(0.01–4.39)	1.10	(0.08–14.12)	1.47	(0.66–3.25)
	Post pubertal	6.52	(0.71–5.93)	0.79	(0.01–53.18)	1.15	(0.02–60.17)	1.92	(0.10–36.48)
BMI									
	Overweight/Obesity	0.28	(0.03–2.17)	3.82	(0.46–31.63)	0.11	(0.01–1.23)	8.39	(0.03–2.38)

Notes: Models adjusted for age (14–16 years), skin color (White), purchasing power (low power), diet (suitable). OR: odds ratio; CI: confidence interval; BMI: body mass index (normal weight), and sexual maturation (prepubescent).

The categories presented in parentheses represent the reference groups.

* p<0.05.

 According to Table 4, in the adjusted analysis, it was observed that female adolescents who reported feeling discriminated against had higher odds of dissatisfaction due to thinness (OR 3.02; 95%CI 1.72–5.31), even after controlling for covariates (age, skin color, purchasing power, diet, physical activity, alcohol consumption, smoking, BMI, and sexual maturation). Physical inactivity was also associated with lower odds of dissatisfaction due to thinness (OR 0.59; 95%CI 0.46–0.75), when compared to physically active female adolescents. Regarding sexual maturation, female adolescents in the pubertal phase had lower odds of dissatisfaction due to thinness (OR 0.54; 95%CI 0.30–0.98) compared with the reference category. In addition, pubertal sexual maturation was associated with lower odds of dissatisfaction due to being overweight (OR 0.42; 95%CI 0.24–0.74) in female adolescents (Table 4). 

**Table 4:** Crude and adjusted multinomial logistic regression analysis between self-reported discrimination and body weight dissatisfaction for females. São José, SC, Brazil – 2019.

		Crude analysis				Adjusted analysis			
		Dissatisfied with thinness		Dissatisfied with being overweight		Dissatisfied with thinness		Dissatisfied with being overweight	
		OR	95%CI	OR	95%CI	OR	95%CI	OR	95%CI
Female									
Feeling of discrimination									
	Yes	2.85	(0.94–8.59)	1.82	(0.68–4.84)	3.02	(1.72–5.31)*	1.65	(0.47–5.69)
Age (years)									
	17–19	0.98	(0.01–6.96)	2.75	(0.03–23.09)	1.30	(0.22–7.59)	1.42	(0.94–2.15)
Skin color									
	Brown	1.38	(0.43–4.36)	1.15	(0.39–3.38)	1.28	(0.51–3.18)	1.29	(0.18–9.09)
	Black	2.73	(0.43–17.39)	1.06	(0.07–14.77)	2.28	(0.52–9.87)	1.07	(0.08–13.90)
	Yellow	1.19	(0.67–2.11)	0.49	(0.11–2.02)	0.96	(0.25–3.63)	1.76	(0.51–6.05)
	Indigenous	0.68	(0.01–70.57)	0.45	(0.10–1.90)	0.41	(0.01–36.15)	0.37	(0.11–1.24)
Purchasing power									
	High	0.07	(0.03–1.40)	0.56	(0.08–3.87)	0.99	(0.27–3.57)	1.52	(0.65–3.56)
Smoking									
	Exposed	0.69	(0.12–3.77)	0.76	(0.01–4.67)	1.37	(0.45–4.14)	0.99	(0.31–3.13)
Alcohol abuse									
	Exposed	4.27	(0.91–19.84)	2.49	(0.04–14.34)	1.56	(0.93–2.63)	1.37	(0.26–7.27)
Balanced diet									
	Inadequate	1.16	(0.42–3.17)	0.61	(0.27–1.40)	0.84	(0.34–2.03)	0.67	(0.12–3.65)
Physically active									
	No	0.69	(0.22–2.16)	1.60	(0.51–4.94)	0.59	(0.46–0.75)*	1.34	(0.01–11.94)
Sexual maturation									
	Pubescent	0.53	(0.06–4.38)	0.74	(0.13–3.95)	0.54	(0.30–0.98)*	0.42	(0.24–0.74)*
	Post pubertal	0.22	(0.10–4.92)	0.19	(0.47–7.92)	0.48	(0.08–2.65)	0.25	(0.02–3.17)
BMI									
	Overweight/Obesity	0.66	(0.03–13.82)	1.44	(0.35–5.81)	1.96	(0.01–86.34)	5.31	(0.61–46.07)

Notes: Models adjusted for age (14–16 years), skin color (White), purchasing power (lower power), diet (suitable). OR: odds ratio; CI: confidence interval; BMI: body mass index (normal weight), and sexual maturation (prepubescent).

The categories presented in parentheses represent the reference groups.

* p<0.05.

## DISCUSSION

 The primary conclusions of this investigation indicated that:

1. Adolescents of both genders who reported feeling discriminated against were dissatisfied due to thinness or dissatisfied due to overweight;
2. Teenage boys who reported feeling discriminated against were more prone to want to decrease their body weight; and
3. Female teenagers who reported feeling discriminated against were more prone to want to increase their body weight.

Experiences of discrimination are associated with higher odds of body weight dissatisfaction among adolescents, regardless of whether dissatisfaction is due to overweight or thinness. These results may be explained by the idealization of body standards promoted by the media, where unattainable and unrealistic beauty ideals are constructed.^21^ In line with previous studies, these findings indicate that discrimination functions as a psychosocial factor that can distort body perception and intensify health-risk behaviors and attitudes, whether through the internalization of aesthetic standards of thinness or muscularity.^22^ Male adolescents who reported experiences of discrimination in the current study showed higher odds of body weight dissatisfaction. These findings could be hypothetically explained, based on prior literature, by increased social media use during adolescence, which has been associated with a higher risk of self-perceived overweight among males and greater body weight dissatisfaction; however, this pathway was not directly assessed in the present study.^23^ Studies indicate that, among males, those who experienced weight-based discrimination reported a stronger desire for thinness, lower body satisfaction, and higher levels of bulimic symptoms compared to those who did not experience such discrimination.^4^

 Female adolescents who reported feeling discriminated against exhibited higher odds of dissatisfaction due to thinness. This finding differs from most previous studies, which have primarily examined experiences of discrimination in relation to overweight-related body dissatisfaction among female adolescents, whereas the present study shows an association between experiences of discrimination and dissatisfaction due to thinness.^11^ This difference may be linked to the coexistence of multiple aesthetic standards: while some adolescents internalize thinness as the ideal, others interpret a "very thin" body as a target of discrimination, which generates an aspiration to gain weight.^24^

 This effect may be amplified by the inherent discrimination faced by females from birth, a practice that often persists into adolescence and adulthood.^25^ These nuances reinforce the idea that weight discrimination should not be analyzed homogeneously. Factors of gender, culture, and social context modulate not only the experience of discrimination but also the direction of its effects on body perception. Although our data reveal significant associations, gaps remain: longitudinal studies in middle-income countries that simultaneously consider discrimination, body perception, and cultural standards of beauty are lacking to understand how these processes intertwine over time. Furthermore, qualitative research could deepen the meanings adolescents attribute to discrimination, especially to understand why some girls interpret it as a stimulus for weight gain, while boys tend to associate it with weight loss. Such evidence is crucial for informing public policies that, in addition to promoting healthy habits, directly address body weight dissatisfaction as a social determinant of health. 

 The strengths of the study include:

1. The application of a validated tool to assess body weight dissatisfaction in the investigated population through;^26^
2. The application of complex sampling methods for statistical analyses, assigning differentiated weights based on the probabilistic selection of sample elements, which considered school size and sampling weight, thus ensuring representative population estimates.^27^

However, the limitations should also be noted, as follows.

1. The study has a cross-sectional design, which does not allow for establishing temporality or causality for the tested associations;
2. It does not assess the accuracy of body weight perception. Our measure captures subjective satisfaction or dissatisfaction with weight status, rather than whether adolescents accurately perceive their anthropometric weight status. Therefore, the findings should not be interpreted as reflecting body image misperception;
3. The study’s internal validity is limited by a 27% sample loss, and its external validity is restricted because data come from a single city;
4. There is no validated material to assess self-reported discrimination among adolescents;
5. The questionnaire used to collect physical activity data did not allow for classification of physical activity levels based on the most recent guidelines;^28^ nevertheless, the physical activity classification in this study^28^ followed the recommendations in effect during the study period;
6. Although BMI is an important indicator of nutritional status, body weight dissatisfaction reflects subjective perceptions that may diverge from objective BMI classifications among adolescents.^29^

Further investigations are needed beyond sex differences regarding the desire to increase or decrease body weight. Other factors, such as discrimination, should be evaluated among subgroups and across multiple structural positions, including race/skin color, ethnicity, weight and gender status.^30^ Further studies are necessary to examine how different forms of discrimination affect individuals. A deeper discussion is required to determine how discrimination affects satisfaction and body weight goals. 

 In conclusion, there is an association between body weight perception and self-reported discrimination among adolescents. Adolescents of both genders who reported feeling discriminated against were dissatisfied due to being thin or overweight. Among male adolescents, those who wished to decrease their body weight were more likely to report feeling discriminated against, while among female adolescents, those who wished to increase their body weight showed higher odds of reporting feeling discriminated against. 

## Data Availability

The database that originated the article is available with the corresponding author.
